# Advances in the automated synthesis of 6-[^18^F]Fluoro-L-DOPA

**DOI:** 10.1186/s41181-021-00126-z

**Published:** 2021-03-10

**Authors:** Ângela C. B. Neves, Ivanna Hrynchak, Inês Fonseca, Vítor H. P. Alves, Mariette M. Pereira, Amílcar Falcão, Antero J. Abrunhosa

**Affiliations:** 1 ICNAS/CIBIT — Institute for Nuclear Sciences Applied to Health, University of Coimbra, Pólo das Ciências da Saúde, Azinhaga de Santa Comba, 3000-548 Coimbra, Portugal; 2 Coimbra Chemistry Center, Chemistry Department, University of Coimbra, Rua Larga, 3004-535 Coimbra, Portugal; 3 Laboratory of Pharmacology, Faculty of Pharmacy, University of Coimbra, Pólo das Ciências da Saúde, Azinhaga de Santa Comba, 3000-548 Coimbra, Portugal

**Keywords:** 6-[^18^F]FDOPA, Automated synthesis, PET, Radiochemistry, Nonproteinogenic amino acid

## Abstract

The neurotracer 6-[^18^F]FDOPA has been, for many years, a powerful tool in PET imaging of neuropsychiatric diseases, movement disorders and brain malignancies. More recently, it also demonstrated good results in the diagnosis of other malignancies such as neuroendocrine tumours, pheochromocytoma or pancreatic adenocarcinoma.

The multiple clinical applications of this tracer fostered a very strong interest in the development of new and improved methods for its radiosynthesis. The no-carrier-added nucleophilic ^18^F-fluorination process has gained increasing attention, in recent years, due to the high molar activities obtained, when compared with the other methods although the radiochemical yield remains low (17–30%). This led to the development of several nucleophilic synthetic processes in order to obtain the product with molar activity, radiochemical yield and enantiomeric purity suitable for human PET studies.

Automation of the synthetic processes is crucial for routine clinical use and compliance with GMP requirements. Nevertheless, the complexity of the synthesis makes the production challenging, increasing the chance of failure in routine production. Thus, for large-scale clinical application and wider use of this radiopharmaceutical, progress in the automation of this complex radiosynthesis is of critical importance.

This review summarizes the most recent developments of 6-[^18^F]FDOPA radiosynthesis and discusses the key issues regarding its automation for routine clinical use.

## Introduction

**Fig. 1:**
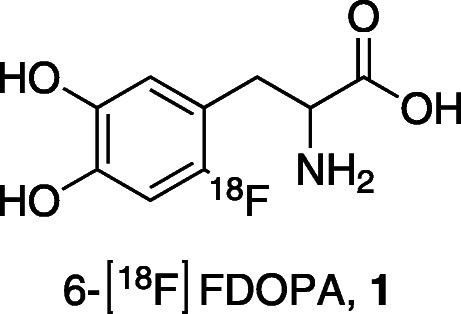
6-[^18^F]FDOPA, 1

The ^18^F-radiolabelled, nonproteinogenic amino acid 3,4-dihydroxy-6-[^18^F]fluoro-*L*-phenylalanine (6-[^18^F]FDOPA, **1**, Fig. 1), has been used, in positron emission tomography (PET) of presynaptic dopaminergic system in the human brain to diagnose several central nervous system disorders such as schizophrenia (Howes et al., 2007; Bose et al., 2008) or Parkinson’s disease (Brooks et al., 2003). The compound has two different enantiomers with *D* or *L-*configuration. As the *d*-isomer of 6-[^18^F]FDOPA presents a lower affinity for blood-brain barrier amino acid transporters, enantiomeric purity is of great importance for PET imaging. Ideally, only the *L*-form should be synthesized (Oldendorf, 1973).

As DOPA is the precursor of the neurotransmitter dopamine, the accumulation of 6-[^18^F]FDOPA **1** in the brain reflects the functional integrity of the presynaptic dopaminergic synthesis and allows us to visualize the conversion of 6-[^18^F]FDOPA **1** in [^18^F]fluorodopamine (Pretze et al., 2014). In 1996, a malignant glioma was found incidentally with 6-[^18^F]FDOPA **1** uptake (Heiss et al., 1996). This finding led to an increased interest in the application of this tracer for oncology, namely in the diagnosis of malignant gliomas (Pretze et al., 2014), neuroendocrine tumours (Neels et al., 2008; Minn et al., 2009; Jager et al., 2008; Balogova et al., 2013; Chondrogiannis et al., 2013), pheochromocytomas (Martiniova et al., 2012; Rischke et al., 2012) and pancreatic adenocarcinomas (Koopmans et al., 2008). Uptake of 6-[^18^F]FDOPA **1** is characteristically high in neuroendocrine cells. These cells store the transported and decarboxylated amines in cytoplasmic neurosecretory granules that vary in size, shape and capacity to store peptide hormones. 6-[^18^F]FDOPA **1** is transported into the neuroendocrine cells via the sodium independent system L, mainly mediated by a large neutral amino acid transporter protein linked to the glycoprotein CD98 (Minn et al., 2009).

Due to its multiple clinical applications, synthesis of 6-[^18^F]FDOPA **1** has become an important issue in radiochemistry. The synthesis has traditionally been quite challenging and the existing processes are complex and typically present low radiochemical yields (Edwards & Wirth, 2015). Several efforts have been made in the development of synthetic processes, which have been reviewed by Wirth et al. and Wängler et al. (Pretze et al., 2014; Edwards & Wirth, 2015). This critical review outlines the recent developments in radiosynthesis of 6-[^18^F]FDOPA **1** as well as its transposition to routine production.

### Synthesis of 6-[^18^F]FDOPA

The development of a suitable automated synthetic process of 6-[^18^F]FDOPA **1** with good radiochemical yield and enantioselectivity, according to Good Manufacturing Practices (GMP), is an issue of great interest in radiochemistry and radiopharmacy. Several methods have been reported in the literature such as isotopic exchange, electrophilic or nucleophilic synthesis and their main results described in the literature so far for each methodology is presented below.

### Isotopic exchange

**Fig. 2:**
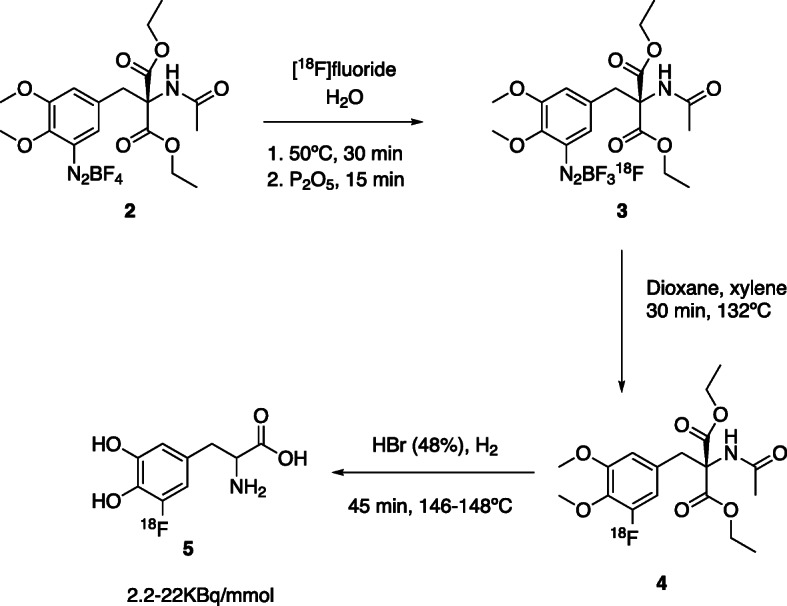
Isotopic Exchange reaction pathway for the synthesis of 5-[^18^F]FDOPA, 5 (Firnau & CSG, 1973)

In 1973, Firnau et al. published the first attempts to synthesize a ^18^F-radiolabelled DOPA derivative, via isotopic exchange (Fig. 2) (Firnau & CSG, 1973). [^18^F]Fluoride was produced in a swimming pool reactor by the ^6^Li(*n*,^4^He)^3^H and ^16^O(^3^H,*n*)^18^F nuclear reactions in a mixture of Li_2_CO in H_2_SO_4_ and H_2_O. Then, the [^18^F]fluoride was distilled twice and the diazonium fluoroborate precursor **2** was added to this solution. After the isotopic exchange reaction, water was removed, and the residue was dried over P_2_O_5_. The residue **3** was then redissolved in dioxane, filtered, and heated to 80 °C. After adding xylene, the solution was heated to 132 °C for 30 min. After solvent evaporation, HBr (48%) was added to hydrolyse **4**, to the final product 5-[^18^F]FDOPA, **5**. The product was obtained with very low molar activity (A_m_) (2.2 to 22KBq/μmol) and very low in vivo stability. In 1984, the same group published the reaction of [^18^F]F_2_ with *L*-DOPA in liquid hydrogen fluoride, yielding a mixture of 2-, 5- and 6-[^18^F]FDOPA of which only 3% was 6-[^18^F]FDOPA, **1** (Firnau et al., 1984). [^18^F]F_2_ was produced from a Ne-target by a tandem Van der Graaff accelerator.

**Fig. 3:**
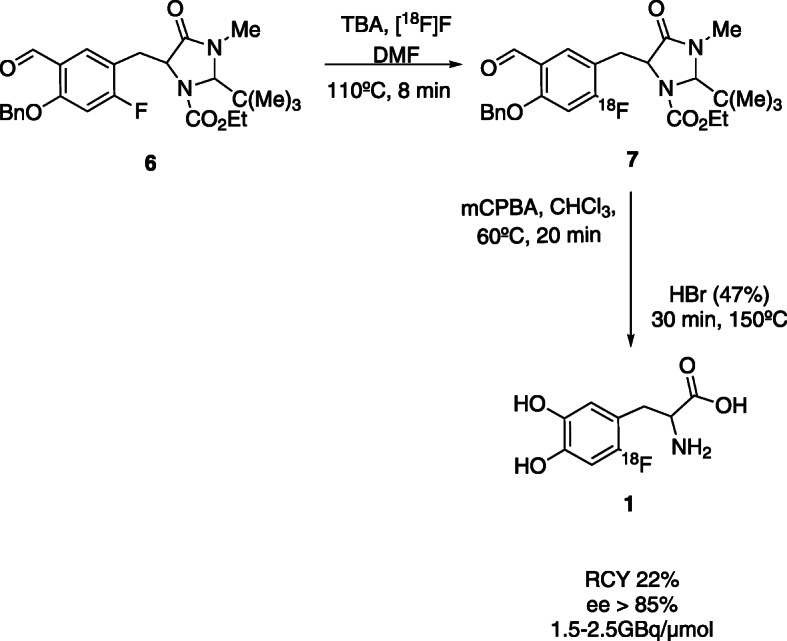
Isotopic Exchange reaction for the synthesis of 6-[^18^F]FDOPA 1 (Wagner et al., 2009)

In 2001, Tierling et al. presented the synthesis of 6-[^18^F]FDOPA **1** by isotopic exchange, with 8–10% of radiochemical yield (RCY) (non-decay corrected (ndc)) and enantiomeric excess (ee) > 85%, in 70 min (Tierlinq et al., 2001). The labelling reaction is based on a carbonyl-activated nucleophilic aromatic substitution of fluorine-19 by fluorine-18, using a benzaldehyde derivative as starting material. Later, Wagner described a similar reaction for radiofluorination of a fluorine-19 precursor with tetrabutylammonium (TBA) [^18^F]fluoride and obtained 6-[^18^F]FDOPA **1** with A_m_ of 1.5–2.5 GBq/μmol and RCYs of 22%, (Fig. 3) (Wagner et al., 2009).

In 2013, Martin’s group automated this method for the GE TRACERLab MX_FDG_. The automated synthesis resulted in 6-[^18^F]FDOPA **1** with reproducible RCY’s between 8 and 12%, in 100 min of reaction, radiochemical purities > 95% and ee >  98% (Rene-Martin et al., 2013).

### Electrophilic method

Later, in order overcome the low radiochemical yield and regioselectivity of the isotopic exchange methods, the synthesis of 6-[^18^F]FDOPA **1** via electrophilic substitution was proposed (Pretze et al., 2014). In this approach, electrophilic fluorination is performed by reacting the precursor with [^18^F]fluorine gas. The main disadvantage of this reaction is the poor RCY and low A_m_ due to the use of carrier added [^18^F]F_2_ gas (Edwards & Wirth, 2015).

Initially, the main route to produce [^18^F]F_2_ for electrophilic fluorination reactions was from the nuclear reaction ^20^Ne(*d*, *α*)^18^F using a F_2_-passivated Ni-target (Nickles et al., 1984). More recently, the ^18^O(*p*,*n*)^18^F nuclear reaction using a ^18^O gas target is the most frequently used as more fluorine-18 is produced (Nickles et al., 1984; Operation, 1995; Hess et al., 2002).

**Fig. 4:**
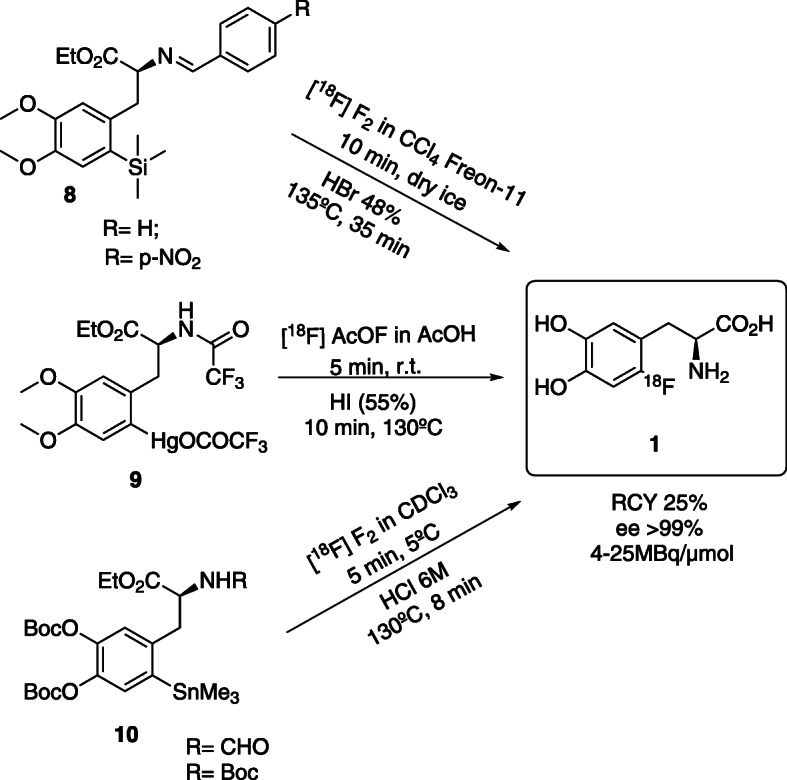
Electrophilic synthesis of 6-[^18^F]FDOPA (Diksic & Farrokhzad, 1985; Adam & Jivan, 1988; Luxen et al., 1990; Bishop et al., 1996; Chaly et al., 1994; Namavari et al., 1992; Dolle et al., 1998; Füchtner et al., 2002; Füchtner & Steinbach, 2003)

The different approaches described in the literature for ^18^F-radiolabelling based on radiodemetallation, desilylation (Diksic & Farrokhzad, 1985), demercuration (Adam & Jivan, 1988; Luxen et al., 1990; Bishop et al., 1996; Chaly et al., 1994) and destannylation (Namavari et al., 1992; Dolle et al., 1998; Füchtner et al., 2002; Füchtner & Steinbach, 2003) are presented in Fig. 4.

Among them, demercuration and destannylation gave the best results and were adapted to automated routine production (Tredwell & Gouverneur, 2012; De Vries et al., 1999). The main route to 6-[^18^F]FDOPA **1**, in this approach, is the reaction of the enantiomerically pure precursors **8**, **9** or **10** with the carrier-added electrophilic fluorine-18, using an automated synthesis module (De Vries et al., 1999; Luxen et al., 1992). Despite the advantages over the previous methods (good ee, > 99% and low reaction times, about 50 min), these reactions present low RCY’s(25% ± 3) and low A_m_ (4 to 25 MBq/ μmol) due to the use of [^18^F]F_2_ which remains the major disadvantage of the electrophilic pathway (Adam et al., 1986).

In 2008, Forsback et al. (Forsback et al., 2008) reported an alternative electrophilic synthesis of 6-[^18^F]FDOPA, **1** were the [^18^F]F_2_ was synthesized in an electrical discharge chamber by a ^18^F/^19^F-exchange reaction. The ^18^F-source was [^18^F]fluoromethane, which was mixed with carrier fluorine in neon (Ne/ 0.5% F_2_) inside the discharge chamber. [^18^F]fluoromethane was produced from methyliodide by a nucleophilic substitution reaction with K [^18^F]F/K_222_ in acetonitrile. 6-[^18^F]FDOPA **1** was obtained with RCYs of 6.4 ± 1.7% (decay corrected) and A_m_ of 3.7 ± 0.9 GBq/μmol. In 2013 Stenhagen et al. (Stenhagen et al., 2013) presented an Ag-mediated electrophilic ^18^F-fluorination of a protected arylboronic ester, which was transformed to a 6-Ag-DOPA derivative with silver triflate. Then, [^18^F]selectfluor bis(triflate) in acetone-d_6_ was added and 6-[^18^F]FDOPA **1** was obtained with RCYs of 19 ± 12% and 2.6 ± 0.3 GBq/μmol in a 20 min reaction.

Although the use of 6-[^18^F]FDOPA **1** as a neurotracer does not necessarily imply high molar activity, for its use in oncology is a very important issue (Koopmans et al., 2005; Kuik et al., 2015). Low molar activities are known to produce pharmacologic effects such as carcinoid crisis by local conversion in the tumour tissue of 6-[^18^F]FDOPA **1** to noradrenaline, induced by aromatic acid decarboxylase and dopamine ß-hydroxylase enzymes (Koopmans et al., 2005), being the major drawback of the electrophilic method.

### Nucleophilic aromatic substitution methods

In order to overcome the limitations of the electrophilic approach, efforts were concentrated on the development of a nucleophilic incorporation of n.c.a. [^18^F]fluoride, which can be obtained with molar activities in order of 314–43,000 GBq/μmol (Edwards & Wirth, 2015; Füchtner et al., 2008) and several synthetic processes have been developed. The most promising processes involve the nucleophilic aromatic substitution of leaving groups such as nitro or trimethylammonium moieties in combination with electron withdrawing groups, with [^18^F]fluoride (Pretze et al., 2014).

The first attempts produced racemic mixtures of the *D*- and *L*- isomers and the pure *L*- isomer was obtained by chiral-HPLC purification, although with a significant loss of activity (Ding et al., 1990; Guillaume et al., 1990).

**Fig. 5:**
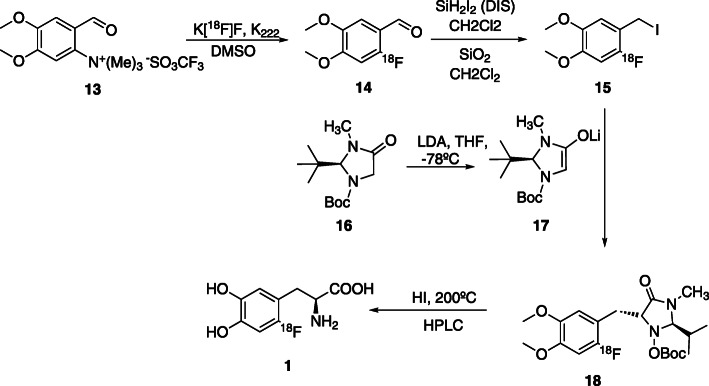
Synthesis of 6-[^18^F]FDOPA **1** starting from aryltrimethylammonium precursor, using chiral auxiliaries (Lemaire et al., 1994)

To avoid this drawback, two alternative approaches were developed. In the first, the reaction starts with the ^18^F-fluorination of an aromatic ring with standard leaving groups in combination with strong electron withdrawing groups (NO_2_ and aldehyde), followed by asymmetric alkylation. In the second, ^18^F-fluorination occurs at a chiral precursor (Pretze et al., 2014; Edwards & Wirth, 2015; Lemaire et al., 1991; Lemaire et al., 1994; Lemaire et al., 1993; Reddy et al., 1993; Najafi, 1995; Horti et al., 1995). Several multistep regioselective nucleophilic synthesis routes of 6-[^18^F]FDOPA **1** have been described in the last years and some results are presented in Table 1. This complex synthesis process normally comprises 5 steps: fluorination, reduction, halogenation, alkylation and hydrolysis. In Fig. 5 we present the reaction steps of this process.

**Table 1:** Synthesis of 6-[^18^F]FDOPA **1** starting from different precursors using chiral auxiliaries

Entry	Precursor	Time (min)	RCY (%)	A_m_ (GBq/μmol)	ee (%)	Ref.
1		110	5–10	n.d.	83–96	(Lemaire et al., 1993)
2		85	6–13	> 74	98	(Najafi, 1995)
3		100–110	12	n.d.	n.d.	(Ding et al., 1990)
4		90	17–29^a^	> 37	> 96	(Lemaire et al., 1994)

Unless otherwise stated, RCYs are given non-decay corrected (ndc). ^a^decay corrected (dc); *n.d*: not determined

The authors (Lemaire et al., 1994) started with the ^18^F-fluorination of the precursor **13**, trimethylammonium veratraldehyde triflate, with 40–70% RCY. This step is favoured by the trimethylammonium triflate, a quaternary salt, which allowed a time reduction by 10 min, when compared with the alternative nitro substitution reaction. The second step is a reductive iodination reaction and the third is an asymmetric inductive alkylation step, which leads to the formation of a new carbon-alpha carbon-beta bond with high diastereoselectivity, all within a total synthesis time of 90 min.

The commercially available nitroveratraldeyde, **11**, was also tested as a precursor for the synthesis of 6-[^18^F]FDOPA **1** by Najafi and Lemaire, using chiral auxiliaries and multistep synthesis (Table 1, entries 1 and 2). However, the product was obtained with low RCY’s (5 to 13%) and the process required quite long reaction times, which is always a great drawback for radiolabelling (Lemaire et al., 1993; Najafi, 1995). Nitropiperonal, **12**, was tested by Ding (Table 1, entry 3) with a similar outcome (Ding et al., 1990). The best results were reported by Lemaire et al. (Table 2, entry 4) which obtained 6-[^18^F]FDOPA **1** with moderate RCY (17–29%) and ee > 96% and A_m_ > 37GBq/μmol (Lemaire et al., 1994).

**Table 2:** Synthesis of 6-[^18^F]FDOPA starting from different precursors using different catalysts

Entry	Precursor	Catalyst	Time (min)	RCY (%)	A_m_ (GBq/μmol)	ee (%)	Ref.
1		21	110	10–15	74–185	95	(Guillouet et al., 2001)
2		21	80–85	7–15*	n.d.	90	(Zhang et al., 2002)
3		21	100	25–30*	n.d.	> 95	(Lemaire et al., 2004)
4		22 and (s)-NOBIN	110–120	16 ± 5	n.d.	96	(Krasikova et al., 2004)
5		21	120	20 ± 4	> 50	≥ 95	(Shen et al., 2009)
6		23/24	63	33–39^a^	> 750	> 97	(Libert et al., 2013)

Unless otherwise stated, RCYs are given non-decay corrected (ndc). ^a^decay corrected (dc); n.d.: not determined

**Fig. 6:**
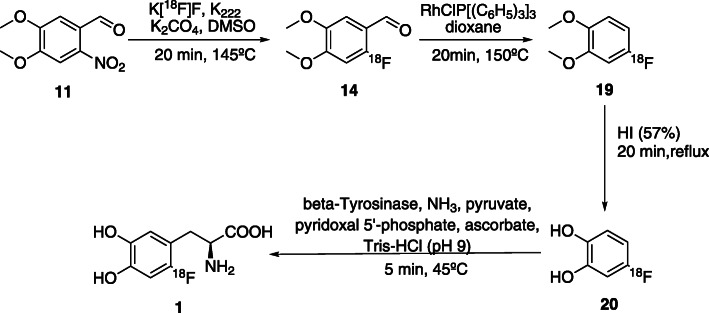
Synthesis of in 6-[^18^F]FDOPA by enzymatic alkylation (Kaneko et al., 1999)

The main disadvantage of this method is the low enantioselectivity as the European Pharmacopoeia (Eur. Ph.) requires the limit for the *D-* enantiomer of 4% (Fluorodopa (^18^F)(prepared by nucleophilic substitution), 2017). In order to solve this problem, Kaneko et al. (Kaneko et al., 1999) proposed an enzymatic reaction step were [^18^F]fluorocatechol was converted in 6-[^18^F]FDOPA **1** with an ee of 100%, A_m_ > 200GBq/μmol within 150 min synthesis time. However, the RCY was only 2%. This synthesis is presented in Fig. 6.

**Fig. 7:**
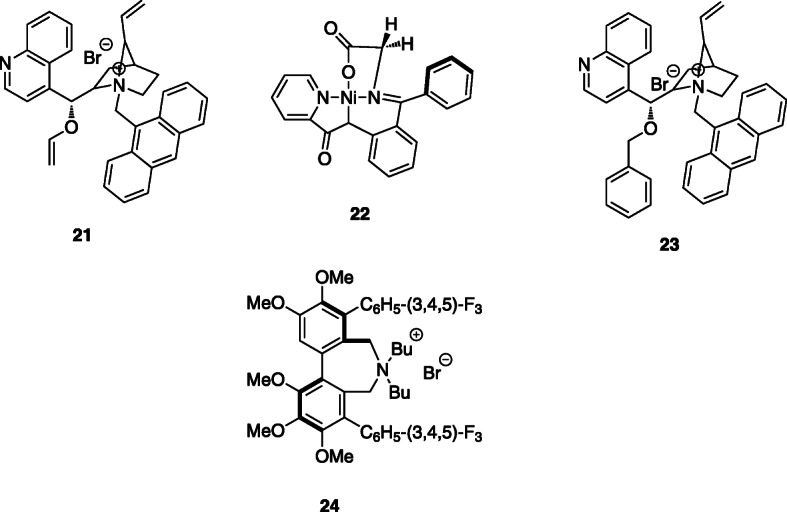
Catalysts. **21**, **22**, **23** e **24**

**Fig. 8:**
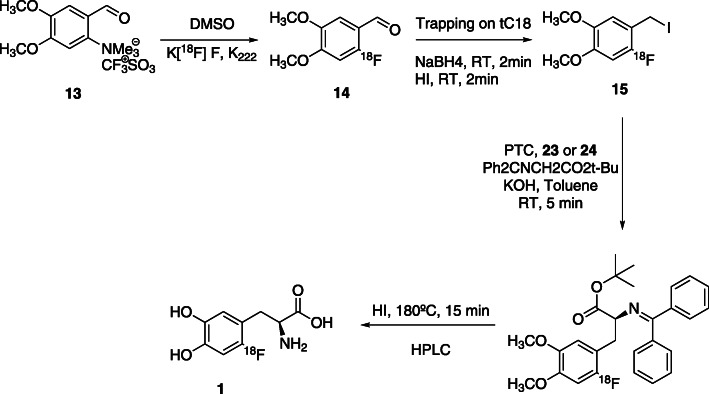
Synthesis of 6-[^18^F]FDOPA starting from trimethylammonium veratraldehyde triflate precursor, using a cPTC strategy (Libert et al., 2013)

In addition, starting from the same precursors, the authors used another strategy which involves the use of chiral phase-transfer catalysts (cPTC) in the asymmetric alkylation key step. In 1997, Corey et al. described the synthesis of O(9)-ally-*N*-(9-anthracenylmethyl)-cinchonidinium bromide **21**, a cPTC used in several asymmetric alkylation reactions (Corey et al., 1997). Usually, the phase-transfer catalyst only allows the enantioselective construction of a new chiral carbon-carbon single bond when the reaction is performed at 0 °C (Lemaire et al., 2004). This is a limitation for the transposition of the process to automation. However, in the last years, a great number of new cPTCs were developed, but only a few of them showed high enantioselectivity at room temperature (Libert et al., 2013). In Fig. 7 we present the structure of the most commonly used cPTCs described so far in the literature and in Fig. 8 we present one example of the use of cPTC.

Table 2 summarizes the literature results for the 6-[^18^F]FDOPA synthesis, starting from the usual precursors but using different catalysts and a Schiff base in the asymmetric alkylation key step.

Guillouet’s group reported the synthesis of 6-[^18^F]FDOPA, using the same precursor **11** and cPTC, yielding the product with RCY of 10–15%, A_m_ of 74–185 GBq/μmol, *ee* of 95% in 110 min (Guillouet et al., 2001). In order to carried out the automation process, they performed the reduction of 6-[^18^F]fluoro-3,4-dimethoxybenzaldehyde with NaBH_4_/H_2_O and the halogenation with gaseous HBr in a Sep-PakC_18_-Plus. The product was eluted from the cartridge with toluene and finally transferred to the alkylation reaction vessel. The reaction was performed at 0 °C in the presence of cPTC **21** and a Schiff base. The acidic hydrolysis was performed at 200 °C during20 minutes, with HI (57%) (Table 2, Entry 1).

In 2002 Zhang et al. (Zhang et al., 2002) also reported a similar multi-step procedure, using trimetylammoniun veratraldehyde triflate **13** as precursor. The process involves the nucleophilic substitution, diiodosilane reductive iodination and phase-transfer catalytic alkylation with cPTC **21** at room temperature, also followed by HI hydrolysis. The product was obtained with low RCY’s of 7–15% (decay corrected) and ee of 90% (Table 2, Entry 2).

Based on previous knowledge, Lemaire’s group (Lemaire et al., 2004) later reported an optimization of the reaction conditions. Starting with the nucleophilic ^18^F-fluorination of trimethylammonium veratraldehyde triflate **13,** followed by the reduction and halogenation (HBr or HI) in a solid support and subsequent alkylation with a Schiff’sbase and cPTC **21**, they obtained 6-[^18^F]FDOPA with 18–30% RCY and ee > 95% (Table 2, Entry 3). Krasikova also prepared 6-[^18^F]FDOPA (Krasikova et al., 2004), using a combination of **22** and *(S)-*NOBIN as a novel substrate/catalyst pair in the alkylation step under mild conditions. 6-[^18^F]FDOPA was obtained with RCY’s of 16% and ee of 96% (Table 2, Entry 4). The disadvantage of this process is the complexity of the catalytic system. Shen et al. presented the synthesis of 6-[^18^F]FDOPA beginning with the ^18^F-fluorination of nitroveratraldehyde **11,** in DMF, followed by the halogenation with freshly prepared diiodosilane. Asymmetric alkylation was performed using cPTC **21** and HBr (48%) or KI were used in the acidic hydrolysis step. The product was obtained with 20 ± 4% RCY and ee ≥ 95% (Table 2, Entry 5) (Shen et al., 2009). The main disadvantage of this method is the instability of diiodosilane used in the reductive iodination of 4,5-dimethoxy-2-[^18^F]fluorobenzaldehyde to 4,5-dimethoxy-2-[^18^F]fluorobenzyliodide.

The best results were reported by Libert et al. in 2013 (Libert et al., 2013). The authors started with the ^18^F-fluorination of precursor **13**, trimethylammonium veratraldehyde triflate. Then the ^18^F-fluorinated aldehyde was trapped on a tC_18_ SPE cartridge where the reduction of the aldehyde and halogenation occurred. Then, the column was eluted with toluene into a reactor where the enantioselective alkylation, in the presence of the cPTC and a prochiral Schiff base, took place. cPTC **23** and **24** (Fig. 8), were tested, yielding enantioselectivities greater than 97%. The last steps were again the hydrolysis with HI at 180 °C for 15 min followed by HPLC purification. The product was obtained with 36% RCY, A_m_ ≥ 753 GBq/μmol and ee of 97%, in a total of 63 min synthesis time (Table 2, Entry 6). The steps of the reaction are presented in Fig. 8.

Multistep reactions, using chiral auxiliaries or cPTC, have proven to solve the problem of enantioselectivity and the best results are within the limits of the pharmacopoeia requirements. Considering the complexity of the process, the main challenge is still the automation, which still requires the development of new, simpler synthetic alternative processes.

### Other fluorination methods

Several alternative approaches for the synthesis of 6-[^18^F]FDOPA have been described. Aromatic nucleophilic substitution (S_N_Ar) with [^18^F]fluoride is a direct method to form the C(sp^2^)-^18^F bond. For this purpose, the precursor typically contains a leaving group and an activating group (electron withdrawing) in the *ortho* or *para* position (Deng et al., 2019). Other alternative strategies have been tested including the ^18^F-fluorination of diaryliodonium salts, the ^18^F-fluorination of spirocyclic iodonium ylides or the transition-metal-mediated aromatic ^18^F-fluorination (with Ni or Cu). In this section, some of these alternative strategies are described (Deng et al., 2019).

**Fig. 9:**
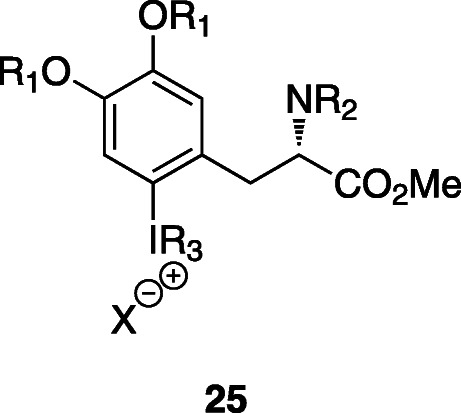
Generic structure of diaryliodonium salts

The strategy usually involves the ^18^F-fluorination of a diaryliodonium salt by ligand exchange, followed by thermal decomposition and hydrolysis, yielding the protected 6-[^18^F]FDOPA. The electron rich aryl groups insure a regioselective ^18^F-fluorination (Edwards & Wirth, 2015). Figure 9 shows the generic structure of this type of precursors.

In Table 3 is summarized the main results reported for 6-[^18^F]FDOPA synthesis using iodonium salts.

**Table 3:** Synthesis of 6-[^18^F]FDOPA using iodonium salts as precursors

Entry	Precursor	Time (min)	RCY (%)	A_m_ (GBq/μmol)	ee (%)	Ref.
1		n.d.	30–40	> 148	> 98	(Edwards & Wirth, 2015)
2		30	5–10	–	n.d.	(Satyamurthy & Barrio, 2010a)
3		20	31 ± 3*	148 ± 74	n.d.	(Ichiishi et al., 2014)

*RCY for protected 6-[^18^F]FDOPA

**Fig. 10:**
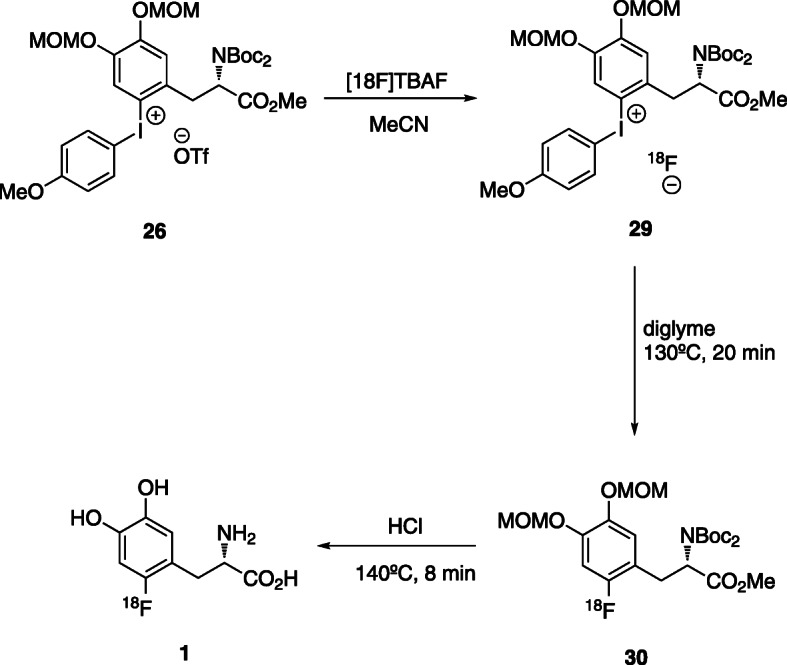
Diaryliodonium triflate precursor for the synthesis of 6-[^18^F]FDOPA by anion exchange followed by thermal decomposition (Edwards & Wirth, 2015)

DiMagno et al. (DiMagno, 2011) patented the synthesis of the diaryliodonium triflate precursor and its application. In the first step, the [^18^F]iodonium fluoride is formed in dry acetonitrile by anion exchange. After removal of salt by filtration, the ^18^F-fluorination of the iodonium fluoride is carried out in a non-polar solvent. The last step is again the acid hydrolysis with HBr 48%. Ground Fluor Pharmaceuticals Inc. (Edwards & Wirth, 2015), reported a similar strategy yielding 6-[^18^F]FDOPA in 30–40% RCY (Table 3, Entry 1). The reaction steps are presented in Fig. 10.

Another approach was based on the use of iodonium ylides for late ^18^F-fluorination stage. Recently, Liang et al. (Rotstein et al., 2014) proposed spirocyclic hypervalent iodine (III) complexes as precursors for one-step regioselective radiofluorination with [^18^F]fluoride. This functionalization shows high efficiency for radiolabelling of a large range of non-activated functionalized arenes and heteroarenes, including some common radiotracers. In 2010 Barrio et al. *(*Satyamurthy & Barrio, 2010b*)* patented the ^18^F-fluorination of a functionalized iodonium ylide, yielding 6-[^18^F]FDOPA in amounts suitable to perform human PET studies. The same authors reported the use of an iodyl precursor **27** that results from the oxidation of the iodine(I) compound, which after ^18^F-fluorination yields 6-[^18^F]FDOPA in 5–10% RCY in 15–30 min (Table 3, entry 2). The explosive nature of the precursor **27** may reduce the utility of this methodology.

Another relevant synthetic approach uses transition-metal-mediated aromatic ^18^F-fluorination. This strategy has proven to be a promising alternative to other methods due to the high reactivity, selectivity and tolerance towards other functional groups.

As an example, Ritter et al. described a nickel-mediated nucleophilic synthesis of protected 6-[^18^F]fluoro-3,4-dihydroxy-*L*-phenylalanine via oxidative ^18^F-fluorination (Lee et al., 2012), Fig. 11.

**Fig. 11:**
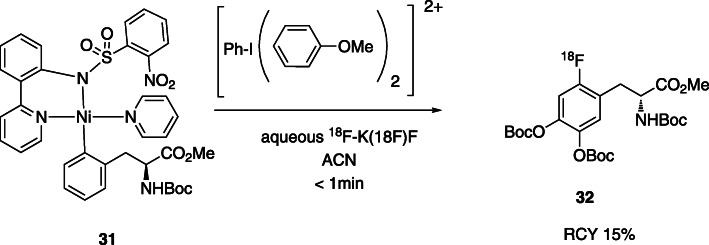
Nickel-mediated synthesis of protected 6-[^18^F]fluoro-3,4-dihydroxy-*L*-phenylalanine (Lee et al., 2012)

The reaction of aqueous [^18^F]fluoride with a nickel complex precursor, in the presence of a hypervalent iodine as oxidant resulted in 15% of product in less than 1 min. The main advantage of this method is the use of aqueous fluoride, avoiding the azeotropic drying steps. However, the iodine oxidant is very unstable and the precursor requires a high complexity synthesis.

Moreover, copper-mediated aromatic radiofluorination has been widely used in aromatic ^18^F-fluorination. In 2014, Scott et al. *(*Ichiishi et al., 2014*)* described a strategy that uses Cu-catalysed radiofluorination of diaryliodonium salts using [^18^F]KF. They tested this method with several molecules, bearing different functional groups, and demonstrated that they can obtain the protected 6-[^18^F]FDOPA with 31 ± 3% RCY and a A_m_ of 148 ± 74% GBq/μmol (Table 3, entry 3).

In 2016, the same group (Makaravage et al., 2016) proposed a cooper-mediated nucleophilic radiofluorination of arylstannanes with [^18^F]KF*.* They tested a range of arylstannes, including TriBoc-*L*-DOPA methyl ester **33** with different reactions times and conditions, including several solvents and additives.

**Fig. 12:**
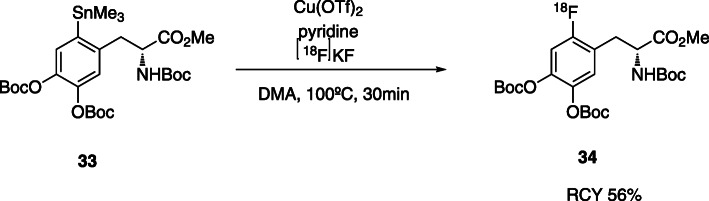
Cooper-mediated synthesis of protected 6-[^18^F]fluoro-3,4-dihydroxy-*L*-phenylalanine (Makaravage et al., 2016)

Starting with 1 equivalent of the commercially available precursor **33** in DMA, in the presence of 2 equivalent of Cu(OTf)_2_ and 15 equivalent of pyridine, the ^18^F-protected FDOPA was obtained in 56 ± 12% yield (Fig. 12). The sensitivity to air and oxygen of this method requires the manipulation of the reagents in a glove box, which makes the automation quite challenging.

**Fig. 13:**
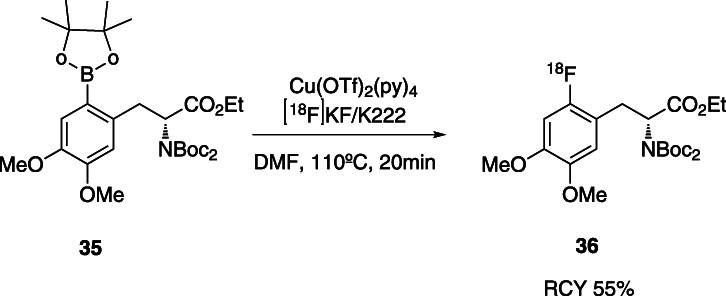
Copper-mediated synthesis of protected 6-[^18^F]fluoro-3,4-dihydroxy-*L*-phenylalanine starting from an aryl boronic derivative precursor (Tredwell et al., 2014)

A similar approach was applied, for the synthesis of 6-[^18^F]FDOPA using copper-mediated ^18^F-fluorination, but using aryl boronic esters as precursors (Tredwell et al., 2014) (Fig. 13).

Recently, Mossine et al. (Mossine, 2019) reported the automation of a copper-mediated, one-pot, high molar activity of 6-[^18^F]FDOPA using a GE TRACERlab MX_FN_.

The advantage of these last methodologies, when compared with the ones of arylstannanes, is the low toxicity of the reagents and higher tolerance to oxygen and air.

As previously stated, routine production of any radiopharmaceutical requires automation and, considering the complexity of the processes described, 6-[^18^F]FDOPA synthesis has been one of the most challenging. Critical aspects such as low radiochemical yields, purities and enantiomeric excesses, sensitivity of reagents and complex manipulations create considerable challenges and, as a consequence, very few methods of synthesis of 6-[^18^F]FDOPA have been successfully automated and used in commercially available modules.

### Automated synthesis

For many years, the only commercially available automated method for the synthesis of 6-[^18^F]FDOPA was the electrophilic destannylation (Tredwell & Gouverneur, 2012; De Vries et al., 1999) described previously. Considering the disadvantages of this method, several efforts have been made to develop an automated nucleophilic synthetic process.

**Fig. 14:**
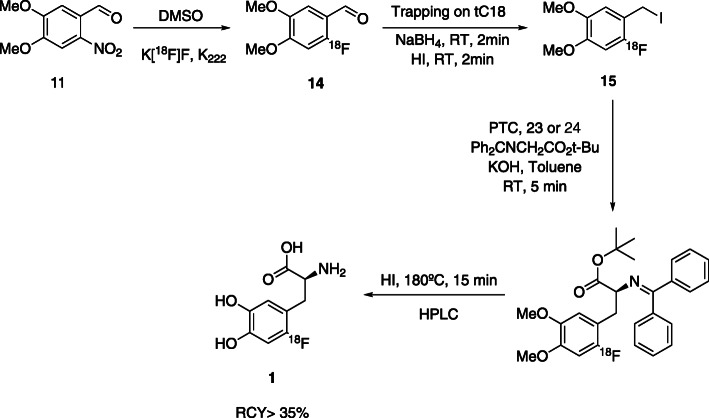
Automated multistep synthesis of 6-[^18^F]FDOPA by Trasis®

Based on developments of Lemaire et al. the cPTC strategy (Lemaire et al., 2004; Libert et al., 2013; Lemaire et al., 2012), was implemented by Trasis® automated module for the synthesis of 6-[^18^F]FDOPA process (Date, n.d.). The multistep synthesis starts from nucleophilic aromatic substitution of nitrobenzaldehyde with [^18^F]fluoride. The activating aldehyde group is then halogenated to iodide followed by enantioselective carbon-carbon bond formation with Schiff’s base, in presence of a cPTC The protected 6-[^18^F]FDOPA is then hydrolysed and purified by semipreparative HPLC, yielding 6-[^18^F]FDOPA with RCYs > 35%, A_m_ 129,5 Gbq/μmol and ee of 97% (Edwards & Wirth, 2015). The process was performed in a Trasis (Ans, Belgium) AllInOne automatic synthesis module cassette-based and provides 6-[^18^F]FDOPA with reproducible results (Edwards & Wirth, 2015), (Fig. 14).

The nucleophilic based method was firstly implemented in 2013 by Martin et al. to a GE (Chicago, Illinois, United States) TRACERlab MX_FDG_ automated module and subsequently commercialized by ABX (Radeberg, Germany) (Rene-Martin et al., 2013). An automated multistep synthesis process, based on SPE cartridges purification was implemented and expanded for other modules, such ORA Neptis® (Philippeville, Belgium) and Siemens (Munich, Germany) Explora™ One, yielding 6-[^18^F]FDOPA with a reported radiochemical purity (RCP) higher than 95% and ee of 98% (Rene-Martin et al., 2013).

**Fig. 15:**
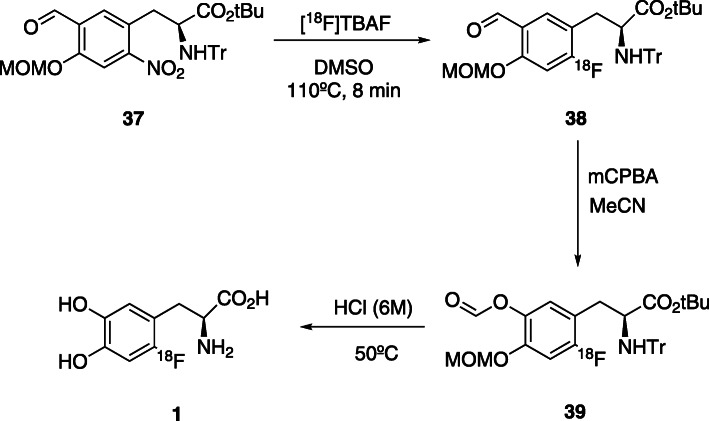
ABX method for automated synthesis of 6-[^18^F]FDOPA (Rene-Martin et al., 2013)

The same process, using the non-carried precursor (ABX 1336) was also developed for an IBA (Louvain-la-neuve, Belgium) module within a set of disposable cassettes (IFP-“Integrated Fluidic Processor”). The synthesis includes, between several steps, trapping, elution and drying of the fluoride, nucleophilic ^18^F-fluorination, oxidation of the intermediate and hydrolysis. The purification is carried out in a set of cartridges yielding the final product formulated in citrate buffer (Fig. 15) with 20 ± 5% RCY and > 99% ee.

This method could be performed in a non-cassette-based system and avoid the semi-preparative purification, which is expensive and time consuming. The main disadvantage is the lower RCY when compared with the Trasis® method. However, both groups are able to routinely produce 6-[^18^F]FDOPA in an automatic synthesis modules.

In both methods, the approach is the direct nucleophilic aromatic substitution. In Trasis® method, the fluorination occurs in nitroveratraldeyde **11**, followed by reduction, halogenation, alkylation, hydrolysis and semi-preparative purification. In the ABX®module, ^18^F-fluorination occurs at a chiral precursor **37** which encompasses an aldehyde as activating group in the *para* position, followed by a Baeyer-Villiger oxidation, in order to transform the aldehyde into an easily hydrolysable group. Despite the differences of the initial precursor molecules, both methods contain a multistep procedure leading to high complexities in the automated processes, with consequent low radiochemical yields.

The same method was performed in an automated model iPHASE FlexLab Module (Australia) by Ya-Yao Huang (Poniger, 2017), yielding 6-[^18^F]FDOPA with RCP > 99%, RCY between 5 and 7% in 110 min.

More recently, based on already commercially available synthetic methodologies, Pretze et al. *(*Pretze et al., 2017*)* evaluated the multistep synthesis based on cPTC and ABX methods using an Eckert&Ziegler (Berlin, Germany) modular-Lab Standard module. The first strategy doesn’t show applicability in this module. However, the second approach, shows better results but, not better than the original performed in a IBA (Louvain-la-neuve, Belgium) module, RCY of 20 ± 1%, A_m_ up to 2.2 GBq/μmol and ee > 96%.

## Conclusions

The high interest of 6-[^18^F]FDOPA as a neurotracer for the diagnosis of central nervous system disorders led to the development of several synthetic processes aiming for the automation for routine production. For this purpose, the automated electrophilic process was implemented and is currently still used. However, when the purpose is the application in diagnosis of other malignancies such as neuroendocrine tumours, pheochromocytoma or pancreatic adenocarcinoma, higher molar activities are required. The low molar activities and radiochemical yields of the electrophilic method are still a great drawback of this process. Therefore, several alternative nucleophilic methods have been developed in the last decades. Direct nucleophilic aromatic substitution was the ideal but, the presence of a good leaving group and also an activation group in *ortho* or *para* positions are required, and only multistep synthesis have been reported so far. To overcome these problems, alternative methods have been developed like ^18^F-fluorination of diaryliodonium salts, spirocyclic iodonium ylides, or transition-metal-mediated ^18^F-fluorination. However, automation remains challenging. Until now, only multistep synthesis have been automated, by Trasis® and ABX®, that due the several steps of reaction and complexity of some steps lead to 6-[^18^F]FDOPA production in low RCY’s concomitantly with time consuming procedures. In summary, we consider that the more recent findings of radiolabelling processes for 6-[^18^F]FDOPA production could be automated and certainly will represent a good alternative to already existent multistep automated processes.

## Data Availability

Data sharing not applicable to this article as no datasets were generated or analyzed during the current study.

## Abbreviations

6-[^18^F]FDOPA: 3,4-dihydroxy-6-[^18^F]fluoro-*L*-phenylalanine
PET: Positron Emission Tomography
GMP: Good Manufacturing Practices
A_m_: Molar activity
RCY: Radiochemical Yield
ndc: Non decay corrected
ee: Enantiomeric excess
TBA: Tetrabutylammonium
DMF: Dimethylformamide
mCPBA: Meta-Chloroperoxybenzoic acid
AcOH: Acetic acid
HI: Hydriodic acid
HCl: Hydrochloric acid
K_222_: Cryptan 2.2.2
HPLC: High-Performance Liquid Chromatography
DMSO: Dimethyl sulfoxide
LDA: Lithium diisopropylamide
THF: Tetrahydrofuran
Boc: Tert-butyloxycarbonyl
dc: Decay corrected
nd: Not determined
Eur. Ph.: European Pharmacopoeia
cPTC: Chiral Phase-transfer catalyst
RT: Room temperature
SPE: Solid phase extraction
SnAr: Aromatic Nucleophilic Substitution
MOM: Methoxymethyl Ether
DMA: Dimethylacetamide

## References

[CR1] Adam MJ, Jivan S (1988). Synthesis and purification of l-6-[^18^F]fluorodopa. Int J Radiat Appl Instrumentation Part A Appl Radiat Isot.

[CR2] Adam MJ, Ruth TJ, Grierson JR, Abeysekera B, Pate BD (1986). Routine Synthesis of L- [^18^F] 6-Fluorodopa with Fluorine- 18 Acetyl Hypofluorite. J Nucl Med.

[CR3] Balogova S, Talbot JN, Nataf V, Michaud L, Huchet V, Kerrou K (2013). ^18^F-Fluorodihydroxyphenylalanine vs other radiopharmaceuticals for imaging neuroendocrine tumours according to their type. Eur J Nucl Med Mol Imaging.

[CR4] Bishop A, Satyamurthy N, Bida G, Barrio JR (1996). Chemical reactivity of the ^18^F electrophilic reagents from the 18O(p,n)^18^F gas target systems. Nucl Med Biol.

[CR5] Bose SK, Turkheimer FE, Howes OD, Mehta MA, Cunliffe R, Stokes PR (2008). Classification of schizophrenic patients and healthy controls using [^18^F] fluorodopa PET imaging. Schizophr Res.

[CR6] Brooks DJ, Frey AK, Marek KL, Oakes D, Paty D (2003). Assessment of neuroimaging techniques as biomarkers of the progression of Parkinson’s disease. Exp Neurol.

[CR7] Chaly T, Bandyopadhayay D, Matacchieri R, Belakhleff A, Dhawan V, Takikawa S (1994). A disposable synthetic unit for the preparation of 3-O-methyl-6-[^18^F]fluorodopa using a regioselective fluorodemercuration reaction. Appl Radiat Isot.

[CR8] Chondrogiannis S, Cristina Marzola M, Al-Nahhas A, Venkatanarayana TD, Mazza A, Opocher G (2013). Normal biodistribution pattern and physiologic variants of ^18^F-DOPA PET imaging. Nucl Med Commun.

[CR9] Corey EJ, Xu F, Noe MC (1997). A rational approach to catalytic Enantioselective Enolate alkylation using a structurally rigidified and defined chiral quaternary ammonium salt under phase transfer conditions. J Am Chem Soc.

[CR10] Date R. AllinOne Specific application manual [ 18 F ] FDOPA. p. 1–52.

[CR11] De Vries EFJ, Luurtsema G, Bru M, Elsinga PH, Vaalburg W (1999). Fully automated synthesis module for the high yield one- pot preparation of 6- [ 18 F ] Fluoro- L -DOPA. Appl Radiat Isot.

[CR12] Deng X, Rong J, Wang L, Vasdev N, Zhang L, Josephson L (2019). Chemistry for positron emission tomography : recent advances in 11C-, 18 F-, 13 N-, and 15 O-labeling reactions. Angew Chem Int Ed.

[CR13] Diksic M, Farrokhzad S (1985). New synthesis of fluorine-18-labeled 6-fluoro-L-dopa by cleaving the carbon-silicon bond with fluorine. J Nucl Med.

[CR14] DiMagno SG. Fluorination of aromatic ring systems. US; US20110313170 A1, 2011.

[CR15] Ding Y-S, Shiue C-Y, Fowler JS, Wolf AP, Plenevaux A (1990). No-carrier-added (NCA) aryl [^18^F]fluorides via the nucleophilic aromatic substitution of electron-rich aromatic rings. J Fluor Chem.

[CR16] Dolle F, Demphel S, Hinnen F, Fournier D, Vaufrey F, Crouzel C (1998). 6-[^18^F]Fluoro-L-DOPA by radiofluorodestannylation : a short and simple synthesis of a new labelling precursor. J Label Compd Radiopharm.

[CR17] Edwards R, Wirth T (2015). 6-fluoro-3,4-dihydroxy- l -phenylalanine - recent modern syntheses for an elusive radiotracer. J Label Compd Radiopharm.

[CR18] Firnau G, Chirakal R, Garnett ES (1984). Aromatic radiofluorination with [^18^F] fluorine gas : 6-[^18^F] fluoro-L-dopa. J Nuci Med.

[CR19] Firnau G, CSG N (1973). [^18^F]5-Fluoro-DOPA with Reactor-Produced Fluorine-18. Int J Appl Radiat Isot.

[CR20] Fluorodopa (^18^F) (prepared by nucleophilic substitution). Injection. In: Pharmeuropa 292; 2017. p. 156–61.

[CR21] Forsback S, Eskola O, Haaparanta M, Bergman J, Solin O (2008). Electrophilic synthesis of 6-[^18^F]fluoro-L-DOPA using post- target produced [^18^F]F2. Radiochim Acta.

[CR22] Füchtner F, Angelberger P, Kvaternik H, Hammerschmidt F, Simovc BP, Steinbach J (2002). Aspects of 6-[^18^F]fluoro-L-DOPA preparation: precursor synthesis, preparative HPLC purification and determination of radiochemical purity. Nucl Med Biol.

[CR23] Füchtner F, Preusche S, Mäding P, Zessin J, Steinbach J (2008). Factors affecting the specific activity of [^18^F]fluoride from a [18O]water target. NuklearMedizin..

[CR24] Füchtner F, Steinbach J (2003). Efficient synthesis of the ^18^F-labelled 3-O-methyl-6-[^18^F]fluoro-L-DOPA. Appl Radiat Isot.

[CR25] Guillaume M, Cantineau R, Chnstiaens L (1990). No-carrier-added regioselective preparation of 6-^18^F-fluoro-L-dopa. J Nucl Med.

[CR26] Guillouet S, Lemaire C, Bonmarchand G, Zimmer L, le Bars D. Large scale production of 6-[^18^F]fluoro-L-DOPA in a semi-automated system. J Labelled Compounds Radiopharm 2001;44:S868–S870.

[CR27] Heiss WD, Wienhard K, Wagner R, Lanfermann H, Thiel A, Herholz K (1996). F-Dopa as an amino acid tracer to detect brain tumors. J Nucl Med.

[CR28] Hess E, Sichler S, Kluge A, Coenen HH (2002). Synthesis of 2- [18 F] fluoro- l -tyrosine via regiospecific. Appl Radiat Isot.

[CR29] Horti A, Redmond DE, Soufer R (1995). No-carrier-added (NCA) synthesis of 6-[^18^F]fluoro-L-DOPA using 3,5,6,7,8,8a-hexahydro-7,7,8a-trimethyl-[6S-(6α, 8α, 8αβ)]-6,8-methano-2H-1,4-benzoxazin-2-one. J Label Compd Radiopharm.

[CR30] Howes OD, Montgomery AJ, Asselin MC, Murray RM, Grasby PM, Mcguire PK (2007). Molecular imaging studies of the striatal dopaminergic system in psychosis and predictions for the prodromal phase of psychosis. Br J Psychiatry.

[CR31] Ichiishi N, Brooks AF, Topczewski JJ, Rodnick ME, Sanford MS, Scott PJH (2014). Copper-catalyzed [ 18 F]fluorination of (Mesityl)(aryl)iodonium salts. Org Lett.

[CR32] Jager PL, Chirakal R, Marriott CJ, Brouwers AH, Koopmans KP, Gulenchyn KY (2008). 6-L-^18^F-fluorodihydroxyphenylalanine PET in neuroendocrine tumors: basic aspects and emerging clinical applications. J Nucl Med.

[CR33] Kaneko S, Ishiwata K, Hatano K, Omura H, Ito K, Senda M (1999). Enzymatic synthesis of no-carrier-added 6-[^18^F]fluoro-l-dopa with β-tyrosinase. Appl Radiat Isot.

[CR34] Koopmans KP, Brouwers AH, De Hooge MN, Van Der Horst-schrivers AN, Kema IP, Wolffenbuttel BH (2005). Carcinoid crisis after injection of 6-^18^F-fluorodihydroxyphenylalanine in a patient with metastatic carcinoid. J Nucl Med.

[CR35] Koopmans KP, Neels OC, Kema IP, Elsinga PH, Sluiter WJ, Vanghillewe K (2008). Improved staging of patients with carcinoid and islet cell tumors with ^18^F-dihydroxy-phenyl-alanine and 11C-5-hydroxy-tryptophan positron emission tomography. J Clin Oncol.

[CR36] Krasikova RN, Zaitsev VV, Ametamey SM, Kuznetsova OF, Fedorova OS, Mosevich IK (2004). Catalytic enantioselective synthesis of ^18^F-fluorinated α-amino acids under phase-transfer conditions using (s)-NOBIN. Nucl Med Biol.

[CR37] Kuik W-J, Kema IP, Brouwers AH, Zijlma R, Neumann KD, Dierckx RAJO (2015). In vivo biodistribution of no-carrier-added 6-^18^F-Fluoro-3,4-Dihydroxy-L-phenylalanine (^18^F-DOPA), produced by a new Nucleophilic substitution approach, compared with carrier-added ^18^F-DOPA, prepared by conventional electrophilic substitution. J Nucl Med.

[CR38] Lee E, Hooker JM, Ritter T (2012). Nickel-mediated oxidative fluorination for PET with aqueous [^18^F] fluoride. J Am Chem Soc.

[CR39] Lemaire C, Damhaut P, Plenevaux A, Comar D (1994). Enantioselective synthesis of 6-[fluorine-18]-fluoro-L-DOPA from no-carrier-added fluorine-18-fluoride. J Nucl Med.

[CR40] Lemaire C, Gillet S, Guillouet S, Plenevaux A, Aerts J, Luxen A (2004). Highly Enantioselective Synthesis of No-Carrier-Added 6-[^18^F]Fluoro-L-dopa by Chiral Phase-Transfer Alkylation. Eur J Org Chem.

[CR41] Lemaire C, Guillaume M, Cantineau R, Plenevaux A, Christiaens L (1991). An approach to the asymmetric synthesis of l-6-[^18^F]fluorodopa via NCA nucleophilic fluorination. Int J Radiat Appl Instrumentation Part A Appl Radiat Isot.

[CR42] Lemaire C, Libert L, Plenevaux A, Aerts J, Franci X, Luxen A (2012). Fast and reliable method for the preparation of ortho- and Para-[^18^F]fluorobenzyl halide derivatives: key intermediates for the preparation of no-carrier-added PET aromatic radiopharmaceuticals. J Fluor Chem.

[CR43] Lemaire C, Plenevaux A, Cantineau R, Christiaens L, Guillaume M, Comar D (1993). Nucleophilic enantioselective synthesis of 6-[^18^F]Fluoro-l-dopa via two chiral auxiliaries. Appl Radiat Isot.

[CR44] Libert LC, Franci X, Plenevaux AR, Ooi T, Maruoka K, Luxen AJ (2013). Production at the curie level of no-carrier-added 6-^18^F-Fluoro-L-Dopa. J Nucl Med.

[CR45] Luxen A, Guillaume M, Melega WP, Pike VW, Solin O, Wagner R (1992). Int J Radiat Appl Instrumentation Part B Nucl Med Biol.

[CR46] Luxen A, Perlmutter M, Bida GT, Van Moffaert G, Cook JS, Satyamurthy N, et al. Remote, semiautomated production of 6-[^18^F]fluoro-l-dopa for human studies with PET. Int J Radiat Appl Instrumentation Part A Appl Radiat Isot. 1990;41(3):275–81. 10.1016/0883-2889(90)90191-i 2158953

[CR47] Makaravage KJ, Brooks AF, Mossine AV, Sanford MS, Scott PJH (2016). Copper-mediated Radiofluorination of Arylstannanes with [^18^F]KF. Org Lett.

[CR48] Martiniova L, Cleary S, Lai EW, Kiesewetter DO, Seidel J, Dawson LF (2012). Usefulness of [^18^F]-DOPA for PET imaging in a mouse model of phechoromocytoma. Nucl Med Biol.

[CR49] Minn H, Kauhanen S, Seppanen M, Nuutila P (2009). ^18^F-FDOPA: A Multiple-Target Molecule. J Nucl Med.

[CR50] Mossine AV, Tanzey SS, Brooks AF, Makaravage KJ, Ichiishi N, Miller JM (2019). One-pot synthesis of high molar activity 6-[18F]fluoro-l-DOPA by Cu-mediated fluorination of a BPin precursor. Org Biomol Chem.

[CR51] Najafi A (1995). Measures and pitfalls for successful preparation of “no carrier added” asymmetric 6-[^18^F]fluor-l-dopa from ^18^F-fluoride ion. Nucl Med Biol.

[CR52] Namavari M, Bishop A, Satyamurthy N, Bida G, Barrio JR (1992). Regioselective radiofluorodestannylation with [^18^F]F2 and [^18^F]CH3COOF: a high yield synthesis of 6-[^18^F]fluoro-l-dopa. Int J Radiat Appl Instrumentation Part A Appl Radiat Isot.

[CR53] Neels OC, Koopmans KP, Jager PL, Vercauteren L, van Waarde A, Doorduin J (2008). Manipulation of [11C]-5-Hydroxytryptophan and 6-[^18^F]Fluoro-3,4-Dihydroxy-L-phenylalanine accumulation in neuroendocrine tumor cells. Cancer Res.

[CR54] Nickles RJ, Daube ME, Ruth TJ (1984). An 18O2 targuet for the production of (^18^F)F2. Int J Appl Radiat Isot.

[CR55] Oldendorf W (1973). Stereospecificity of blood-brain barrier permeability to amino acids. Am J Phys.

[CR56] Operation T (1995). Development of An Improved Target for [^18^F]. F2 Production.

[CR57] Poniger SS (2017). Automated Nucleophilic Radiosynthesis of [^18^F] FDOPA with a modified iPHASE FlexLab Module Ya-Yao Huang, Stan Poniger, Chia-Ling Tsai. 22nd ISRS.

[CR58] Pretze M, Franck D, Kunkel F, Foßhag E, Wängler C, Wängler B (2017). Evaluation of two nucleophilic syntheses routes for the automated synthesis of 6-[^18^F]fluoro-L-DOPA. Nucl Med Biol.

[CR59] Pretze M, Wängler C, Wängler B (2014). 6-[^18^F]fluoro-L-DOPA: a well-established neurotracer with expanding application spectrum and strongly improved radiosyntheses. Biomed Res Int.

[CR60] Reddy GN, Haeberli M, Beer HF, Schubiger AP (1993). An improved synthesis of no-carrier-added (NCA) 6-[^18^F]Fluoro-l-DOPA and its remote routine production for PET investigations of dopaminergic systems. Appl Radiat Isot.

[CR61] Rene-Martin BD, Huebner S, Juettler S, Saul S, Clausnitzer A (2013). First SPE Method for Routine Production of Nucleophilic (^18^F)-L-DOPA. J Label Compd Radiopharm.

[CR62] Rischke HC, Benz MR, Wild D, Mix M, Dumont RA, Campbell D (2012). Correlation of the genotype of Paragangliomas and Pheochromocytomas with their metabolic phenotype on 3,4-Dihydroxy-6-^18^F-Fluoro-L-Phenylalanin PET. J Nucl Med.

[CR63] Rotstein BH, Stephenson NA, Vasdev N, Liang SH (2014). Spirocyclic hypervalent iodine(III)-mediated radiofluorination of non-activated and hindered aromatics. Nat Commun.

[CR64] Satyamurthy N, Barrio J. No-carrier-added nucleophilic [f-18] fluorination of aromatic compounds. Wo 2010117435 A2, 2010a.

[CR65] Satyamurthy N, Barrio J. No-carrier-added nucleophilic [f-18] fluorination of aromatic compounds. Wo 2010117435 A3, 2010b.

[CR66] Shen B, Ehrlichmann W, Uebele M, Machulla H-J, Reischl G (2009). Automated synthesis of n.c.a. [^18^F]FDOPA via nucleophilic aromatic substitution with [^18^F]fluoride. Appl Radiat Isot.

[CR67] Stenhagen ISR, Kirjavainen AK, Forsback SJ (2013). Fluorination of an arylboronic ester using [^18^F]selectfluor bis(triflate): application to 6-[^18^F]fluoro-l-DOPA. Chem Commun.

[CR68] Tierlinq T, Hamacher K, Coenen HH, Gmbh FJ (2001). A new Nucleophilic asymmetric synthesis. J Label Cpd Radiopharm.

[CR69] Tredwell M, Gouverneur V (2012). ^18^F labeling of arenes. Angew Chem Int Ed.

[CR70] Tredwell M, Preshlock SM, Taylor NJ, Gruber S, Huiban M, Passchier J (2014). A general copper-mediated Nucleophilic ^18^F fluorination of Arenes. Angew Chem Int Ed.

[CR71] Wagner FM, Ermert J, Coenen HH (2009). Three-step, “one-pot” Radiosynthesis of 6-Fluoro-3,4-Dihydroxy-L-phenylalanine by isotopic exchange. J Nucl Med.

[CR72] Zhang L, Tang G, Yin D, Tang X, Wang Y (2002). Enantioselective synthesis of no-carrier-added (NCA) 6-[^18^F]fluoro-l-DOPA. Appl Radiat Isot.

