# Correction to: Advances in the automated synthesis of 6-[^18^F]Fluoro-L-DOPA

**DOI:** 10.1186/s41181-021-00132-1

**Published:** 2021-05-20

**Authors:** Ângela C. B. Neves, Ivanna Hrynchak, Inês Fonseca, Vítor H. P. Alves, Mariette M. Pereira, Amílcar Falcão, Antero J. Abrunhosa

**Affiliations:** 1 ICNAS/CIBIT — Institute for Nuclear Sciences Applied to Health, University of Coimbra, Pólo das Ciências da Saúde, Azinhaga de Santa Comba, 3000-548 Coimbra, Portugal; 2 Coimbra Chemistry Center, Chemistry Department, University of Coimbra, Rua Larga, 3004-535 Coimbra, Portugal; 3 Laboratory of Pharmacology, Faculty of Pharmacy, University of Coimbra, Pólo das Ciências da Saúde, Azinhaga de Santa Comba, 3000-548 Coimbra, Portugal

**Correction to: EJNMMI Radiopharm Chem 6, 11 (2021)**

**https://doi.org/10.1186/s41181-021-00126-z**

Following publication of the original article (Neves et al. 2021), some errors were identified in the article text:

-Page 3, **Fig. 2**, where it reads **1** it should read **5** and ^18^F should be at position 5 of the aromatic ring.

-Page 6, where it reads “*MA > 96%”*, it should read “A_m_ > 37GBq/μmol*”*

-Page 7, where it reads “D- enantiomer of 2%” it should read “D- enantiomer of 4%”

-Page 8, where it reads “*using a cPTC*” it should read: *“using different catalysts”.*

-In page 8, **Fig. 7**, caption, where it reads *“Chiral phase transfer catalysts (cPTC),”*, it should read *“Catalysts****.****”*

-Pages 8–9, where it reads “*6-[*^*18*^*F]fluoro-3,4-dimethoxybenzyl bromide”* and *“Sep-PakC*_*18*_*-Plus reactor.”*, it should read *“6-[*^*18*^*F]fluoro-3,4-dimethoxybenzaldehyde”* and *“Sep-PakC*_*18*_*-Plus”, respectively.*

-Page 9, **Fig. 8**, where it reads “**22** or **23**”, it should read “**23** or **24**” and, number **24** under the structure, should be disregarded.

-Page 9, where it reads *“Krasikova also prepared nitropiperonal* ***12*** *(Krasikova et al., 2004), using a combination of cPTC,* ***22****.*” it should read *“Krasikova also prepared 6-[*^*18*^*F]FDOPA (Krasikova et al., 2004), using a combination of* ***22****.*”

-Page 10, caption of Table 2, where it reads *“using cPTC as alkylation agent.”* it should read *“using different catalysts.”*

-Page 10, Table 2, line 1, where it reads “PTC”, should read “catalyst”.

-Page 12, Table 3, entry 3, where it reads “31 ± 3”, it should read “31 ± 3*”, and where it reads “n.d. not described”, it should read “n.d. not described. *RCY for protected 6-[^18^F]FDOPA”.

-Page 13, where it reads, “6-[^18^F]FDOPA with 31 ± 3% RCY” it should read *“the protected 6-[*^*18*^*F]FDOPA with 31 ± 3% RCY”*.

-Page 14, where it reads *“described in section 2.2.”*, it should read *“described previously”*.

-Page 15, **Fig. 14** where it reads “**13**, **14, 22 or 23**”, it should read “**14**, **15, 23 or 24**” and number **24** under the structure should be disregarded.

-Page 15, where it reads “MA 129500 Gbq/μmol”, it should read “A_m_ 129,5 GBq/μmol”.

-Page 15, **Fig. 15,** where it reads “**31**, **32** and **33”,** it should be read “**37, 38** and **39**”.

-Page 16, where it reads “nitroveratraldeyde **12”**, it should read nitroveratraldeyde **11**″ and, where it reads “precursor **31”** it should read precursor **37**″.

-There are multiple occurrences of [18F]. They should read [^18^F].

- MA is used throughout for Molar Activity. It should be A_m_. 

-The following reference is missing: Mossine A V., Tanzey SS, Brooks AF, Makaravage KJ, Ichiishi N, Miller JM, et al. One-pot synthesis of high molar activity 6-[18F]fluoro-l-DOPA by Cu-mediated fluorination of a BPin precursor. Org Biomol Chem. 2019;17(38):8701–5.

The original article (Neves et al. 2021) has been corrected.
